# The Cleveland Clinic Experience with Supraclavicular and Popliteal Ambulatory Nerve Catheters

**DOI:** 10.1155/2014/572507

**Published:** 2014-04-02

**Authors:** Ramez Gharabawy, Alaa Abd-Elsayed, Hesham Elsharkawy, Ehab Farag, Kenneth Cummings, Gamal Eid, Maria Mendoza, Loran Mounir-Soliman, Richard Rosenquist, Wael Ali Sakr Esa

**Affiliations:** ^1^Anesthesiology, Cleveland Clinic, 9500 Euclid Avenue, E-31, Cleveland, OH 44195, USA; ^2^Anesthesiology Department, University of Cincinnati, 231 Albert Sabin Way, Cincinnati, OH 45267-0531, USA; ^3^Anesthesiology Department, University of Wisconsin School of Medicine and Public Health, 600 Highland Avenue, Madison, WI 53792-3272, USA; ^4^CCLCM of Case Western Reserve University, Cleveland Clinic, 9500 Euclid Avenue, E-31, Cleveland, OH 44195, USA; ^5^Anesthesia and Outcome Research, Department of General Anesthesia and Pain Management, Cleveland Clinic, 9500 Euclid Avenue, E-31, Cleveland, OH 44195, USA; ^6^Outcome Research Department, Anesthesia Institute, Cleveland Clinic, 9500 Euclid Avenue, E-31, Cleveland, OH 44195, USA; ^7^Pain Management Department, Anesthesia Institute, Cleveland Clinic, 9500 Euclid Avenue, E-31, Cleveland, OH 44195, USA

## Abstract

Continuous peripheral nerve blocks (CPNB) are commonly used for intraoperative and postoperative analgesia. Our study aimed at describing our experience with ambulatory peripheral nerve catheters. After Institutional Review Board approval, records for all patients discharged with supraclavicular or popliteal catheters between January 1, 2009 and December 31, 2011 were reviewed. A licensed practitioner provided verbal and written instructions to the patients prior to discharge. Daily follow-up phone calls were conducted. Patients either removed their catheters at home with real-time simultaneous telephone guidance by a member of the Acute Pain Service or had them removed by the surgeon during a regular office visit. The primary outcome of this analysis was the incidence of complications, categorized as pharmacologic, infectious, or other. The secondary outcome measure was the average daily pain score. Our study included a total of 1059 patients with ambulatory catheters (769 supraclavicular, 290 popliteal). The median infusion duration was 5 days for both groups. Forty-two possible complications were identified: 13 infectious, 23 pharmacologic, and 6 labeled as other. Two patients had retained catheters, 2 had catheter leakage, and 2 had shortness of breath. Our study showed that prolonged use of ambulatory catheters for a median period of 5 days did not lead to an increased incidence of complications.

## 1. Introduction

Continuous peripheral nerve blocks (CPNB) are often used to provide intraoperative and postoperative analgesia. Effective pain control after painful orthopedic procedures may facilitate earlier patient discharge, improve acute rehabilitation, and increase patient satisfaction. It has been shown that the use of CPNB decreases the use of intravenous (IV) and oral opioids, improves rehabilitation, and decreases length of hospital stay [1–3].

In addition, CPNB were found to provide more potent analgesia than wound catheters and fewer undesirable effects compared to epidural infusion [4]. The development of safe electronic infusion pumps for ambulatory use has improved the feasibility of discharging patients with perineural catheters. It is common practice to leave perineural catheters* in situ* for a limited period of time (2-3 days) [5, 6]. However, at Cleveland Clinic, ambulatory CPNB are routinely used for a longer period of time with no observed increase in the incidence of complications and with earlier patient mobilization and rehabilitation.

In this retrospective study, we describe our experience with ambulatory CPNB in regard to infection and pharmacological complications.

## 2. Methods

After Cleveland Clinic Institutional Review Board approval, records for all patients discharged with supraclavicular or popliteal catheters between January 1, 2009 and December 31, 2011 were reviewed. Data collection was performed by investigators from the electronic medical record.

All catheters were inserted using a standard technique. Blocks were performed by a staff anesthesiologist assisting a trainee (resident/fellow). Both physicians, as well as the ancillary personnel (block room nurses and/or technicians), were wearing a new hat and mask for each patient. Both physicians practiced hand wash and removed hand watches, bracelets, and jewelry before putting on sterile gloves. Sterile gowns were not used.

The skin was cleansed with chlorhexidine gluconate in isopropyl alcohol; then a sterile drape was applied and the skin was cleaned for a second time with chlorhexidine. All catheters (Arrow, StimuCath continuous, nerve block procedural kit ASK 05060-cch 19 Ga, 60 cm catheter, insulated needle, 18 g 3.81 inch) were inserted using an in-plane ultrasound technique under strict aseptic conditions with the ultrasound probe covered with a sterile sheath. The catheter was advanced 3–5 cm beyond the needle tip. During supraclavicular catheter insertion, the catheters were placed dorsolateral to the nerve plexus. During popliteal catheter insertion, the catheters were placed next to the nerve with the needle coming from the lateral side of the thigh. The catheter was advanced 3–5 cm beyond the tip of the needle to end within the space between the semitendinosus and semimembranosus muscles medially and biceps femoris muscle laterally.

All catheters were tunneled under the skin, a sterile adhesive and chlorhexidine-impregnated patch were applied around the catheter site, and then the site was covered with clear occlusive dressing. All patients received infusions using the AmbIT pump (Summit Medical Production, Inc., Salt Lake city, UT, USA). We chose this pump as it is technically easy for patients to use and adjust. After catheter placement, an initial bolus dose of 20 mL ropivacaine 0.75% was administered. All patients were evaluated for sensory and motor block prior to surgery. Before discharge, the catheters were connected to AmbIT pumps infusing ropivacaine 0.2% with an 8 mL/hour basal rate and a 12 mL demand dose once per hour. In addition, patients were given a prescription for oxycodone 5 mg every 4 hours with acetaminophen 500 mg (1-2 tablets) every 8 hours; both were to be used as needed for pain for five days. After meeting the discharge criteria, patients with ambulatory catheters were discharged home. Patients needed to have access to a phone to be reached daily, and needed an access to a nearby emergency facility if urgent care was needed.

A licensed practitioner (physician assistant or registered nurse) provided verbal and written discharge instructions to the patients, (Appendix A). The correct use of the infusion pump controls was demonstrated, with repeat demonstration by the patient with family members present. Daily follow-up phone calls were conducted by an Acute Pain Service member, in which they recorded pain scores, signs or symptoms of infection, and pharmacological complications. The rate of infusion was adjusted daily as needed based on the pain score by instructing the patient to reprogram the pump to the desired infusion rate, (Appendix B). Patients either removed their catheters at home with real-time simultaneous telephone guidance by a member of the Acute Pain Service or had them removed by the surgeon during a regular office visit.

On the fifth day, patients were instructed to stop the infusion for 6 hours and then remove the catheter if their pain scores were less than 5 and well tolerated by the patients. If pain was more than or equal to 5 we asked patients to restart their infusions and we did the same every day until the catheter was removed. The primary outcome of this analysis was the incidence of which were categorized as pharmacologic, infectious, or other, for example, retained catheter. The secondary outcome measure was the average daily verbal response pain score.

The patients were compared with basic descriptive statistics by catheter type. Variables of interest included patient demographics, surgery location and type, and infusion duration. Categorical variables are presented as number (percent). Continuous variables are presented as medians with interquartile ranges. R version 2.12.0 (The R Foundation for Statistical Computing, Vienna, Austria).

Any adverse neurologic symptom reported by a patient was listed as a pharmacological complication, regardless of type or severity. Any sign or symptom of infection at the catheter site (erythema, drainage, or swelling) was labeled as an infectious complication. Any other complications such as a retained catheter were labeled as other.

## 3. Results

A total of 1059 patients with ambulatory catheters (769 supraclavicular, 290 popliteal) were reviewed. The median infusion duration was 5 days for both groups.  Table 1 describes patient characteristics and results.

Forty-two complications were identified: 13 were infectious (11 in the supraclavicular group and 2 in the popliteal group), 23 were pharmacologic (22 in the supraclavicular group and 1 in the popliteal group), and 6 were labeled as other. Tables 2, 3, and 4 describe these patients. Two patients had retained catheters which were removed surgically; these were looped around the nerve without actual knotting. Two patients had catheter leakage and two had shortness of breath (one due to pneumonia and the other due to pulmonary embolism and myocardial infarction).

All infections were superficial, presenting as redness and tenderness at the catheter site. In all cases, patients were instructed to remove the catheter. In only one case did the patient require antibiotics; the remainder of the infections resolved without any intervention other than catheter removal.

Pharmacological complications consisted of ringing in the ears, ipsilateral numbness, hoarseness, and significant ptosis. All pharmacological complications resolved after discontinuing the infusion for 2-3 hours or removing the catheter without any sequelae. Most of the pharmacological complications presented in patients with supraclavicular catheters.

The time-weighted average for daily verbal pain scores (0–10) at the time of telephone contact for patients with supraclavicular or popliteal catheters was 2, showing overall satisfactory postoperative pain control.

## 4. Discussion

Expanded use of regional anesthesia has increased patients' benefit in terms of better control of intraoperative and postoperative pain, increased patient satisfaction, decreased postoperative nausea and vomiting, and early mobilization and rehabilitation.

One of the major concerns regarding the use of CPNB has been the potential for complications, such as catheter site infection, nerve injury, and local anesthetic toxicity. A recent meta-analysis evaluating 19 studies showed that major complications were rare. The most frequent minor complication attributable to peripheral nerve block was excessive motor block [2]. The most common technical difficulties have been related to pump malfunction, catheter misplacement, displacement, obstruction, and catheter migration [2, 3, 6].

The frequency of infection associated with peripheral nerve catheters remains poorly defined. Recent studies have shown that between 23 and 57% of peripheral nerve catheters become colonized, but only 0–3% result in localized infection and less than 1% result in serious infections [7]. The 1.2% infection rate in our study is consistent with the reported rates, although the infusion durations were considerably longer. Severe infectious complications reported in the literature include psoas abscess complicating continuous femoral nerve blocks [8, 9], axillary abscess and necrotizing fasciitis after single shot and continuous axillary nerve blocks [10, 11], and thigh and interscalene abscesses after continuous popliteal, sciatic, and interscalene nerve blocks, respectively [12, 13]. The American Society for Regional Anesthesia and Pain Medicine (ASRA) guidelines highlight the importance of asepsis in regional anesthesia procedures, mainly during needle and catheter insertion, specifically hand washing, the use of protective barriers (mask, gloves, gowns, and drapes) and chlorhexidine-containing skin disinfectants [14]. Guidelines for practice improvement must be built according to specific actual risk applied to each procedure and certainly cannot be extrapolated without some restrictions. CPNB are increasing in popularity, and the incidence of infections associated with CPNB is rare. Pharmacological complications (including neurological symptoms) associated with CPNB are rare. A review of the literature showed that the incidence of neurological symptoms 6 months after the block is 0.6%, with most of the symptoms due to causes unrelated to the block [15]. Capdevila et al. [9] reported an incidence of 6.6% in adult population and Ganesh et al. [5] reported an incidence of 1.6%. In the study by Ganesh et al., 108 children were discharged home with ambulatory catheters; the authors reported prolonged numbness (>24 hours) to be the most common complication noticed and it happened in 3 patients [5]. They also reported that numbness resolved spontaneously without any consequences [5]. In our study the incidence of pharmacological complications including neurological complications was 2.2% (2.9% in the supraclavicular group and 0.3% in the popliteal group); most of them were excessive numbness of the blocked limb and all resolved within 24 hours without any residual deficit. The low incidence of pharmacological complications in our study and their short duration may be due to the use of ultrasound in placing our catheters and confirming that the medications are infusing around the nerve and not intraneurally. Two patients had retained catheters which were removed surgically and they found to be looped around the nerve without actual knotting. There were no specific difficulties with the insertion of these two catheters which were threaded the usual 5 cm beyond the tip of the needle. Both catheters were successfully removed by surgical exploration with no complications after radiological localization of the catheters. Knotting of peripheral nerve catheters is rare, occurring in only 0.13% of patients in a retrospective review [16], but it represents the most reported cause for catheter retention in the literature.

A review of the literature shows few cases describing difficulty in removing peripheral nerve catheters mostly secondary to knotting or excessive advancement under the skin, as previous investigators have demonstrated a relationship between length of catheter advancement and subsequent knotting [17]. Considering the multiple catheter knots reported with insertion >5 cm, and the lack of data suggesting insertion lengths >5 cm is beneficial, recommending a maximal insertion of 5 cm seems warranted [4]. Patients with new onset shortness of breath should go to the emergency department as this may be due to coincidental comorbidities in rare occasions, which if not discovered and treated in a timely fashion would be life threatening. Shortness of breath was present in 2 of our patients who had supraclavicular catheters. Both patients were instructed to go to the emergency department. The first was found to have pneumonia and the second was found to have a myocardial infarction and pulmonary embolism. Two other patients had leakage from the catheter port and this was resolved by tightening the connection between the catheter port and the tubing which was performed by a physician in the emergency department.

Despite the rise in popularity of continuous regional techniques for ambulatory surgery, little has been studied regarding patient perception of the technique. Retrospective surveys have shown that patients are generally satisfied with ambulatory perineural infusions including the removal of catheters themselves [18].

## 5. Conclusion

The results of our study demonstrate that the prolonged use of ambulatory catheters for a period up to 5 days did not lead to an increased incidence of complications as compared to other studies. Our main complications were minor infections and pharmacological symptoms, which resolved with catheter removal and without the need for additional medical intervention. Patients who presented to the emergency department with shortness of breath had other underlying comorbidities such as myocardial infarction and pulmonary embolism. Vigilance in dealing with patients with ambulatory catheters is crucial to prevent complications.

## Figures and Tables

**Table 1:** Supraclavicular and popliteal catheters.

Catheter type	Surgery site	Procedure	Age (y)	Sex	Duration (days)	Average pain score^†^	Infection	Pharmacologic complication	Other complications
Supraclavicular *N* = 769	Shoulder 498 Elbow 63 Arm 87 Wrist 88 Hand 33	Arthroplasty∗ 257 ORIF 128 Arthroscopy 148 Rotator cuff 87 Tendon repair 19 Other^‡^ 130	57 [47, 66]	M 410 F 359	5 [4, 6]	2 [1, 4]	11 (1.4%)	22 (2.9%)	6 (0.8%)

Popliteal *N* = 290	Leg 16 Ankle 198 Foot 76	Arthrodesis 138 ORIF 69 Osteotomy 32 Arthroplasty 11 Other^§^ 40	53 [41, 63]	M 103 F 187	5 [4, 7]	2 [1, 3]	2 (0.7%) Total: 1.2%	1 (0.3%) Total: 2.2%	0 (0%) Total: 0.6%

M: male; F: female.

Categorical variables are presented as number of patients.

Continuous variables are presented as median (interquartile range).

∗“Arthroplasty” includes total and hemiarthroplasty.

^†^“Average pain score” is the time average of daily verbal pain scores (0–10) at telephone contact.

^‡^“Other” (*N* < 10 each) includes closed reduction, external fixation, exploration, debridement, hardware removal, nerve transposition, osteotomy, and arthrodesis.

^§^“Other” (*N* < 10 each) includes incision and drainage, Achilles' tendon repair, and toe amputation.

ORIF: open reduction and internal fixation.

**Table 2:** Infectious complications.

Age	Sex	Catheter	Duration (days)	Symptoms	Treatment	Comorbidities
42	F	Popliteal	4	Swelling and drainage	Resolved with catheter removal	Thigh abscess

61	F	SC	2	Redness and swelling	Resolved with catheter removal	Hyperlipidemia

52	M	SC	13	Redness and tenderness	Instructed to remove the catheter and see his surgeon, symptoms resolved with no intervention	Hypertension, DM

76	F	SC	4	Redness and swelling	Resolved with catheter removal	Steroid treatment

43	F	SC	2	Redness and swelling	Resolved with catheter removal	Hypertension, hyperlipidemia

35	F	SC	4	Redness and swelling	Resolved with catheter removal	Anemia

38	M	SC	3	Blisters underneath the dressing, redness at the insertion site	Catheter removed in ED, symptoms were resolved within 2 days	None

46	M	SC	5	Redness and tenderness	Resolved with catheter removal	Hypertension, DM, seizures

56	M	SC	7	Redness, swelling, and tenderness	Resolved with catheter removal	None

63	F	SC	3	Redness and purulent discharge	Removed in ED, one dose of IV daptomycin, and oral linezolid	Gastritis, irritable bowel syndrome

60	F	Sc	5	Blisters underneath the dressing and redness	Resolved with catheter removal	None

45	F	Popliteal	6	Redness and tenderness	Resolved with catheter removal	Hypertension, DM

63	M	SC	4	Redness, swelling at the site, nodule 1 inch from the site	CT Of the neck in ED showed no fluid collection, no antibiotics, symptoms resolved within few days	CRPS, hypertension, chronic renal disease, seizures,

SC: supraclavicular catheter; COPD: chronic obstructive pulmonary disease; DM: diabetes mellitus; CT: computerized tomography; CRPS: complex regional pain syndrome; ED: emergency department.

**Table 3:** Pharmacological complications.

Age	Sex	Catheter	Duration (day)	Symptoms	Treatment	Comorbidities
23	F	Popliteal	4	Ringing in the ears with the initial injection	Resolved completely with no intervention	None

22	F	SC	5	Ipsilateral ptosis	Resolved with holding the infusion for 2 hours	None

73	M	SC	2	Hoarseness	Resolved after catheter removal	Hypertension

48	M	SC	7	Ipsilateral ptosis	Resolved with pump off, returned with infusion, resolved with catheter removal	Hypothyroidism

72	F	SC	7	Numbness of ipsilateral hand	Resolved with catheter removal	None

51	M	SC	3	Ringing in the ears, started at home	Instructed to hold the infusion, but the ringing persists, instructed to remove the catheter, and it was resolved completely	Psoriasis

51	M	SC	5	Hoarseness, ipsilateral numbness, and weakness of the hand	Improved with holding the infusion, resolved with catheter removal	Obesity, DM, smoking

38	F	SC	4	Ipsilateral ptosis	Resolved with catheter removal	Hypothyroidism, smoking

29	M	SC	3	Ipsilateral ptosis and fingers tingling	Resolved with catheter removal	Hyperlipidemia

45	M	SC	4	Ringing and numbness in ipsilateral ear	Resolved with holding the infusion	None

75	F	SC	8	Ipsilateral ptosis and facial hyperemia	Resolved with decreasing the concentration to 0.1% ropivacaine	Rheumatoid arthritis, hypertension, colon cancer, and breast cancer

45	M	SC	3	Ipsilateral ptosis	Improved with holding the infusion, resolved with catheter removal	Obstructive sleep apnea and coronary artery disease

65	M	SC	5	Ipsilateral numbness of the face	Improved with holding the infusion, resolved with catheter removal	Hypertension, gout

38	M	SC	3	Tingling and numbness of ipsilateral fingers	Improved with holding the infusion, resolved with catheter removal	None

42	F	SC	4	Ipsilateral ptosis, stuffy nose, and metallic taste in mouth	Improved with holding the infusion, resolved with catheter removal	None

43	M	SC	4	Ipsilateral ptosis	Improved with holding the infusion, resolved with catheter removal	Osteoarthritis

56	M	SC	7	Weakness and numbness of ipsilateral hand	Gradually improved, resolved with catheter removal	None

65	F	SC	6	Hoarseness	Resolved with catheter removal	Hypertension, anxiety

49	F	SC	5	Hoarseness	Resolved with catheter removal	None

60	F	Sc	5	Hoarseness, ipsilateral ptosis	Improved with holding the infusion, resolved with catheter removal	Anxiety, hypothyroidism

56	F	SC	3	Numbness of ipsilateral fingers	Resolved with catheter removal	Obesity, hypertension, obstructive sleep apnea, and DM

45	F	SC	6	Ipsilateral ptosis and numbness of fingers	Resolved with catheter removal	None

39	F	SC	5	Ipsilateral ptosis	Resolved with catheter removal	Anxiety

**Table 4:** Other complications.

Age	Sex	Catheter	Duration (days)	Symptoms	Treatment	Comorbidities
30	F	SC	3	Retained catheter	Removed in ED by surgical extraction at bed side	Depression

62	M	SC	6	Retained catheter	Removed in ED by slight traction with no complication	Coronary artery disease (CAD)

68	F	SC	5	Small amount clear leakage at catheter insertion site	Resolved with reinforced dressing	Osteoarthritis, hypertension

57	M	SC	4	Leakage from catheter tubing	Catheter found to be disconnected at the hub, in ED catheter cleaned with chlorhexidine, cut with sterile scissors, sterile hub applied	Hypertension

70	M	SC	10	Shortness of breath (SOB)	Patient advised to turn off the pump and to go to hospital, chest X-ray showed pneumonia, started on antibiotics, catheter removed	CAD, hypertension

62	F	SC	3	SOB, dizziness, and sweating	Patient advised to turn off the pump and go to the emergency department. Found to have myocardial infarction and pulmonary embolism. Catheter removed. Subsequently discharged home	Hypertension, DM

## Appendices

### A. AmbIT Pump (discharge instructions)

#### A.1. Home Going Instructions

Your surgeon and anesthesiology pain management team have determined that a continuous peripheral nerve block is an option for pain management following your surgical procedure. This information is provided for you regarding the outpatient management of the AmbIT pump.

(1) The peripheral nerve block catheter and infusion pump are intended to help reduce your postoperative pain. Not all surgical pain may be relieved by this method of pain control. Therefore, you will likely need oral pain medication prescribed by your surgeon. Please carefully follow the directions for these oral pain medications.
(2) The local anesthetic medication infusing via the AmbIT pump will produce some degree of numbness in the intended surgical area supplied by those nerves. Due to this numbness it is imperative that you remain protective of your surgical limb from heat, pressure, chemicals, or other objects to avoid injury.
(3) You will likely have some degree of muscle weakness in your arm, hand, leg, or foot from the effect of the local anesthetic. Do not support yourself or bear weight on the arm, hand, leg, or foot while the local anesthetic nerve block is infusing.
(4) There are no narcotics in the solution.
(5) This medication will not interfere with any medications you are currently taking. It will also not interfere with any pain medications you have ordered.
(6) The pump does not require height for infusion as opposed to an IV infusion. The solution and AmbIT pump are placed in a fanny pack for your convenience.
(7) Please note that it is important to keep the dressing over the catheter clean and dry. Do not change the dressing. NO SHOWERS.

##### A.1.1. How to Use the Pump

(1) This pump is disposable and is to be thrown out. It is not reusable.
(2) Left over solution can be disposed of down the sink.
(3) The AmbIT pump runs on 2 AA batteries. New batteries have been placed in the pump.
(4) The AmbIT pump makes a noise while it is infusing, a type of “grinding noise.” It will make this noise about every 20–30 seconds while the pump is on.
(5) When you give yourself a bolus dose the AmbIT pump will make a loud continuous grinding noise for about 10 min. This is normal, when the bolus dose is complete the noise will stop.
(6) The AmbIT pump has already been preset with rates.
  *Basal Rate*. This is the continuous rate of the medication per hour.
  *Bolus Dose*. This is for the moment when you need an extra dose of the anesthetic medication. You can give yourself a bolus dose every hour if needed. If you do not need the extra dose there is no need to press the bolus dose button. You cannot overdose yourself; the pump will only give you 1 bolus dose an hour.
(7) This is the Run/Pause button.
(8) This is the bolus dose button.
(9) When the green light is blinking on the bolus dose button it means that the AmbIT pump is on and working.
(10) The screen on the pump reads with a decimal point; it will look like this 136.5 mL. That number continues to count up as the solution infuses. It is designed to turn off when the number reaches 1000.0 mL.

##### A.1.2. Taking Out the Catheter

Before removing the catheter, make sure that the AmbIT pump has been off for 6–8 hours. We want all of the numbness to be gone and normal sensation returned. If normal sensation has not returned please call the Acute Pain Service.

(1) Wash your hands.
(2) Remove all of the tape.
(3) Gently pull on the catheter; it is in about 5-6 inches. It should come out easily.
(4) If there is resistance or you cannot pull the catheter out, cover the site back up with the provided Tegaderm and call the Acute Pain Service.
(5) After catheter removal, the site may bleed a small amount; this is normal. You may hold pressure over the catheter site for 5–10 minutes and then apply a band aid to the area. The band aid may be removed later in the day.
(6) Notify the Acute Pain Service for any pain, redness, continued bleeding, or drainage from the catheter insertion site and also notify for persistent numbness or weakness in the arm, hand, leg, or foot following the catheter removal.

##### A.1.3. When to Call the Acute Pain Service

Please contact the Acute Pain Service if you have any questions or notice the following symptoms during or following the nerve block infusion.

(1) Increase in pain.
(2) Redness, tenderness, swelling, or drainage at the nerve block catheter insertion site.
(3) Lightheadedness, dizziness, or sedation.
(4) Blurred vision.
(5) Ringing in your ears, metallic taste in your mouth, numbness, or tingling around your mouth.
(6) Any shortness of breath.
(7) Difficulty in swallowing.
(8) Drowsiness.
(9) Confusion.
(10) Any discoloration (redness, bluish color changes) of the hand, fingers, foot, or toes.

#### A.2. Contact Information

##### A.2.1. Acute Pain Service

(1) Acute Pain Service number: (number was provided here).
If no response call:
(2) Consultant: (number is provided here).
If no response call:
(3) The Cleveland Clinic Foundation operator at (number was provided here) and ask for consultant pager (number was provided here).

##### A.2.2. Trouble Shooting the Pump

(1) If the pump shows* MA* on the screen try replacing the batteries, if that does not work call the Acute Pain Service.
(2) If the pump shows* OCL*, there is a kink somewhere in the tubing. Make sure that the tubing clamps are open and moving freely. Also ensure that you are not lying or sitting on the tubing.

### B. Daily Phone Encounter

#### B.1. Supraclavicular Catheter Documentation following Phone Call

Day of surgery:
Type of surgery:
Catheter site:
Solution:
Rate of infusion:
Called and talked to patient, states pain level is:
Patient states the catheter dressing is (intact or specify if otherwise) and denies: (redness, fever, draining, edema, pain, or specify if otherwise).
Patient denies metallic taste in mouth, ringing in ears, dizziness, shortness of breath, hoarseness of voice, or specify if otherwise.
Patient is able to move fingers: Yes or No.
Comments. Patient is satisfied with pain control, will continue with pump, or specify if otherwise.

#### B.2. Popliteal (Sciatic) Catheter Documentation following Phone Call

Day of surgery:
Type of surgery:
Catheter site:
Solution:
Rate of infusion:
Called and talked to patient, states pain level is:
Patient states the catheter dressing is (intact or specify if otherwise) and denies: (redness, fever, draining, edema, pain or specify if otherwise).
Patient denies metallic taste in mouth, ringing in ears, dizziness, shortness of breath, hoarseness of voice, or specify if otherwise.
Patient is able to move toes: Yes or No.
Comments. Patient is satisfied with pain control, will continue with pump, or specify if otherwise.
